# The Effects of Presurgical Nasoalveolar Molding on the Midface Symmetry of Children with Unilateral Cleft Lip and Palate: A Long-term Follow-up Study

**DOI:** 10.1097/GOX.0000000000001764

**Published:** 2018-07-09

**Authors:** Wasmiya A. AlHayyan, Sharat Chandra Pani, Aziza J. AlJohar, Fawzi M. AlQatami

**Affiliations:** From the * Cleft Clinic, Amiri Hospital, Kuwait City, Kuwait; † Riyadh Colleges of Dentistry and Pharmacy, Riyadh City, Kingdom of Saudi Arabia; ‡ Pediatric Dentistry, Riyadh College of Dentistry and Pharmacy (RCDP), Riyadh, Kingdom of Saudi Arabia; § Cleft Lip Craniofacial Program KFSHRC, Satalite Pediatric Dentistry Clinic Children Cancer Center (KFCCC), Riyadh, Kingdom of Saudi Arabia.

## Abstract

**Background:**

Midface symmetry is an important indicator of success of complete unilateral cleft lip and palate (CUCLP) treatment. There is little literature on the long-term effects of presurgical nasoalveolar molding (PNAM) on midface symmetry in children treated for CUCLP. This study aimed to compare children with CUCLP who underwent PNAM before surgical interventions, children who did not receive PNAM, and age- and sex-matched controls in terms of midface symmetry.

**Methods:**

We evaluated 39 frontal facial photographs of 13 patients with CUCLP who underwent PNAM as part of the treatment (group 1: PNAM), 13 patient with CUCLP who did not undergo PNAM (group 2: no nasoalveolar molding), and 13 age- and sex-matched controls. The children were evaluated in their fifth year of life. Three midline and 3 bilateral orthopometric midface landmarks were programmed using a custom software (OnyxCeph3, Image Instruments GmbH, Germany), and corresponding linear measurements from the midline were obtained and compared between the groups using 1-way analysis of variance and Scheffe’s post hoc test.

**Results:**

Significant differences were observed between the control and CUCLP groups for the measurements of the proanasale, subnasale, and zygion. However, there were no significant differences between the PNAM and no nasoalveolar molding groups for the 6 midface landmarks.

**Conclusions:**

PNAM does not seem to significantly impact the long-term midface symmetry in children with CUCLP.

## INTRODUCTION

Oral cleft is the most common craniofacial anomaly in the world, present in 1 of every 700 live births.^1^ Despite multiple advances in the treatment of oral cleft, challenges remain in achieving satisfactory functional and aesthetic outcomes. Facial deformities associated with oral cleft not only lead to functional impairments but also to aesthetic deficiencies mainly manifested in the nose and upper lip regions.^2^

The theoretical foundation of cartilage molding is based on the fact that high levels of estrogen result in high levels of hyaluronic acid at birth, which in turn increases the elasticity of cartilage, allowing the fetus to pass through the birth canal.^3^ The presurgical nasoalveolar molding (PNAM) method is used in intraoral orthopedics for shaping of the alveolus and nose of patients with oral clefts.^4^ The proposed advantages of PNAM include improved placement of cleft segments, which allows surgical closure with minimal scaring, better reported aesthetic outcomes, and facilitation of feeding and speech.^5–7^ Critics of the technique have, however, pointed out that PNAM is an expensive and complex approach^8^ with no effect on the maxillary arch and occlusion.^9^

One of the main long-term advantages claimed by proponents of PNAM is improved nasal symmetry and lip appearance,^10,11^ and facial symmetry.^12^ However, few long-term follow-up studies have investigated whether changes in facial symmetry are retained as the child grows. The midface has been documented as the region most affected after cleft-lip surgery.

A consequence of the policy of standardized protocols at medical centers is that a study designed to compare protocols must be conducted at multiple centers. The cleft center in Kuwait was established in 1991 at Amiri Hospital, Kuwait City. The Grayson treatment approach using PNAM as a presurgical orthopedic treatment has been used in this center since 2008. In contrast, the treatment protocol at King Faisal Specialist Hospital Riyadh, Saudi Arabia, does not include the use of PNAM. This study examined the records of children with cleft lip and palate in their fifth year of life (4–5 years of age) to evaluate changes in midface symmetry. Groups of children who did and did not undergo PNAM before treatment of complete unilateral cleft lip and palate (CUCLP) were then compared with each other and age- and sex-matched controls. The study supposed a null hypothesis that there would be no difference in the facial asymmetry of children treated with PNAM before surgery when compared with those who had not received PNAM.

## MATERIALS AND METHODS

### Research and Ethical Approval

The study proposal was registered with the research center of the Riyadh Colleges of Dentistry and Pharmacy (RCsDP), Riyadh, Saudi Arabia, and an ethical approval was obtained from the institutional review board of RCsDP and Al-Amiri Hospital, Kuwait City, Kuwait, and of King Faisal Specialist Hospital and Research Centre, Riyadh city, Saudi Arabia. Patient confidentiality was maintained using the protocols of the above-mentioned hospitals, and written informed consent was obtained from the parents before the use of any pictures or records for analysis.

### Patient Recruitment

The records of patients treated between June 2009 and December 2013 at Al-Amiri Hospital, Kuwait city, Kuwait, and King Faisal Specialist Hospital and Research Center, Riyadh, Saudi Arabia, were retrieved from the databases of the centers. Parents of children who had completed 4 years of follow-up were contacted, and informed consent was obtained for their participation in the study.

**Fig. 1.**
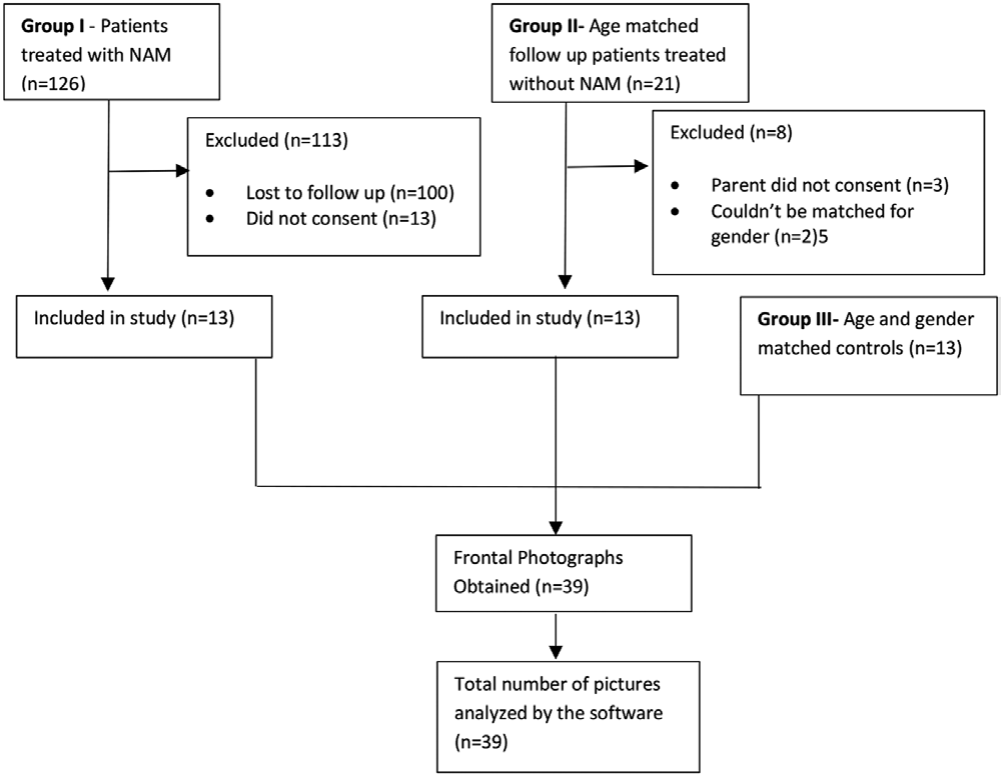
Sample selection and analysis.

Two study groups and 1 control group were formed. Group 1 (PNAM group) included patients with CUCLP who received PNAM as a part of their oral cleft treatment protocol at the Al-Amiri Hospital, Kuwait City, Kuwait, whereas group 2 [no nasoalveolar molding (NNAM) group] comprised patients with CUCLP who did not undergo PNAM before oral cleft repair at King Faisal special hospital, Riyadh City, Kingdom of Saudi Arabia. Thirteen patients in the PNAM group, and 21 patients in the NNAM group met the selection criteria. Thirteen patients with CUCLP from the NNAM group and 13 controls (with no history of oral cleft) were age- and sex-matched to the children in the PNAM group, yielding a final sample size of 39 (Fig. 1).

### Evaluation of Midfacial Symmetry

**Fig. 2.**
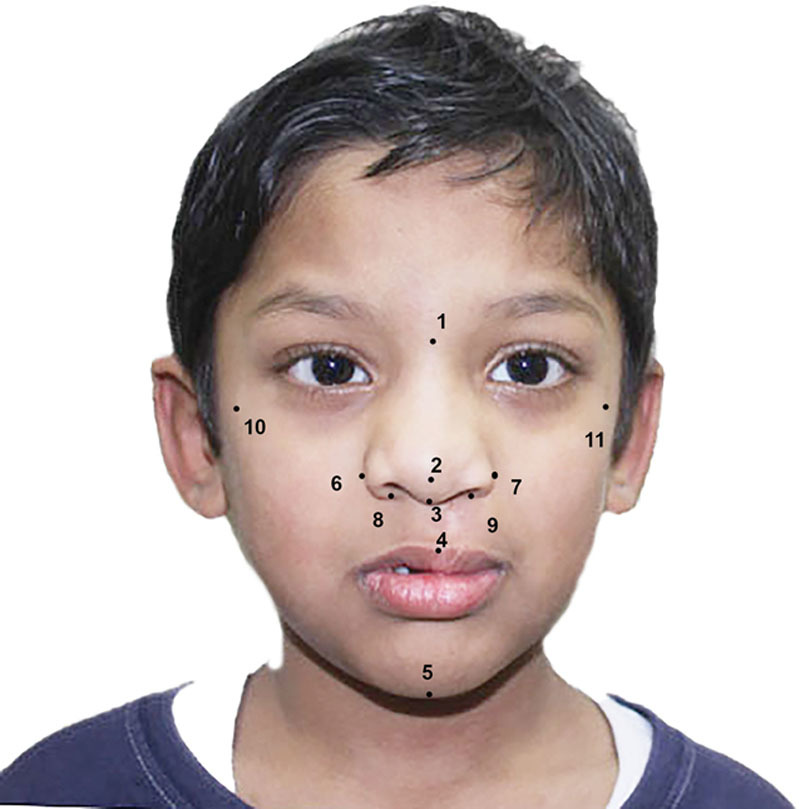
Midface landmarks analyzed in the study.

A frontal photograph of the patient was taken by positioning the patient with the Frankfort horizontal plane parallel to the floor using a digital single lens reflex camera (N5000, Nikon Corp. Tokyo, Japan) from a distance of 5 feet. The images were then standardized to ensure the cleft side was on the right of the patient (Fig. 2). Eight previously used landmarks^13^ were programmed and analyzed using a custom digital dental imaging software program (OnyxCeph3, Image Instruments GmbH, Germany). An imaginary line from the nasion to the gnathion was used to determine the midline (Fig. 2). Linear measurements of the remaining 6 landmarks were obtained from the midline. Three unilateral and 3 bilateral facial landmarks (Table 1) were employed to evaluate midfacial symmetry using 2-dimensional frontal digital photographic images obtained using previously published guidelines (Fig. 2).^14^ These measurements were performed by a single examiner (W.A.). Intraexaminer calibration was carried out by repeating the analysis of 10 control photographs after an interval of 1 week. For all landmarks, the mean length from the midline was compared among the different groups.

**Table 1.**
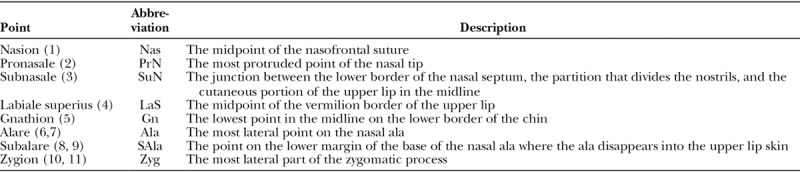
Points Analyzed in the Study

### Statistical Analyses

Descriptive statistics were tabulated, and the chi-square test was used to determine significance of differences between groups. Intraexaminer reliability of the landmarks was tested using Cronbach’s alpha. Pearson’s chi-square was used to evaluate the significance of differences for parametric variables such as sex, cleft side, or side of deviation of midline landmarks. Differences in deviation of the unilateral landmarks from the constructed midline and differences between the cleft side and noncleft side for the bilateral landmarks were compared between the groups using 1-way analysis of variance (ANOVA) and Scheffe’s post hoc test. All statistical analyses were carried out using the SPSS 22 data processing software (IBM Corp, Armonk N.Y.). The level of statistical significance was set to *P* < 0.05.

## RESULTS

Of the 26 patients with CUCLP included in the study, the majority had the cleft on the left side (n = 19). Although there were more boys (n = 24) than girls (n = 15), the difference was not statistically significant (chi-square = 1.232, *P* = 0.454). The intraexaminer reliability evaluated using the interclass correlation coefficient was good for all the landmarks, with Cronbach’s alpha ranging from a high of 0.975 (pronasale) to a low of 0.774 (subnasale; Table 2).

**Table 2.**
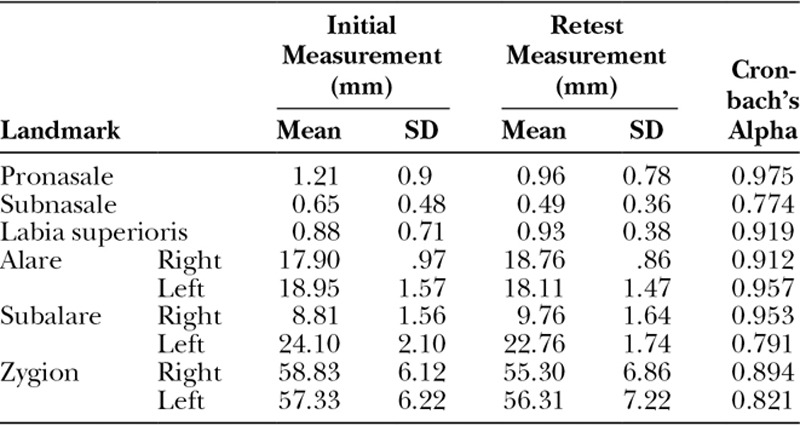
Intraexaminer Reliability of the Landmarks Measured

No significant differences were observed in the side of deviation for the midline landmarks (Table 3). For the pronasale and subnasale unilateral landmarks, there were significant differences between the groups in the distance from the midline, whereas no significant difference was observed for the labia superioris (Table 4). Scheffe’s post hoc test revealed significantly greater deviations for the pronasale in the PNAM and NNAM groups compared with the control group (*P* < 0.05), whereas there was no significant difference between the PNAM and NNAM groups (*P* = 0.087). For the subnasale, there was a significant difference between the PNAM group and the control group (*P* < 0.05) but no significant differences between the PNAM and NNAM groups (*P* = 0.568), and between the NNAM and control groups (*P* = 0.222). For the alare and subalare, there were no significant differences between the groups on the noncleft side. However, significant differences existed between the groups on the cleft side. Scheffe’s post hoc test revealed that while a significant difference (*P* < 0.05) existed between the control group and both the PNAM and NNAM groups, there was no significant difference between the PNAM and NNAM groups (*P* = 0.892). No significant differences were observed among the groups for zygion values on both the cleft and noncleft side (Table 5).

**Table 3.**
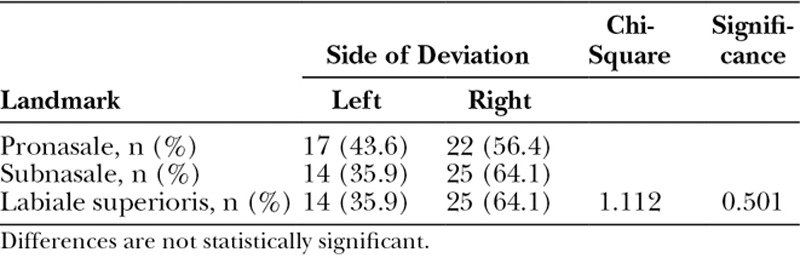
Distribution of the Side of Deviation of Unilateral Landmarks

**Table 4.**
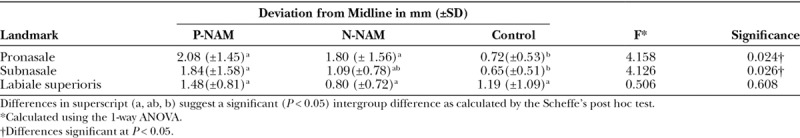
Deviation of the Midline Landmarks from the Constructed Midline

**Table 5.**
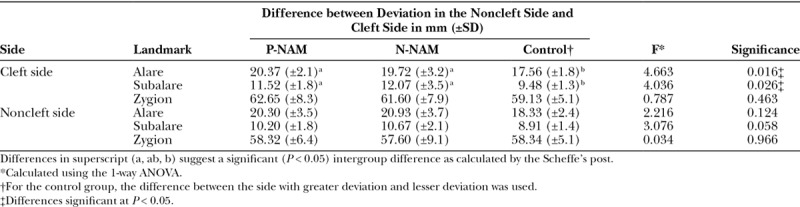
Significance of Difference of Bilateral Landmarks on the Cleft and Noncleft Sides

## DISCUSSION

The role of PNAM in the reduction of postcleft asymmetry has been a matter of controversy in the literature.^15,16^ Although immediate postsurgical benefits of PNAM are evident and potential long-term positive effects of PNAM in CUCLP have been proposed,^12,17,18^ the lack of studies of long-term effects of PNAM complicates the understanding of the role of underlying muscular tensions and the surgery itself in the shaping of the face.^19^ This study evaluated the effects of PNAM on midface symmetry 4 years after surgery.

We found significant differences between the patients with cleft and control participants with respect to the pronasale (*P* = 0.025) and subnasale (*P* = 0.024), which is in agreement with previous long-term studies.^12^ However, there were no significant differences between the PNAM and NNAM groups.

A similar lack of significance for the bilateral landmarks supports the view that while PNAM may facilitate the surgical closure of the cleft lip, there is no sufficient evidence to definitively demonstrate long-terms benefits of the technique.^16^

Most of the criticism of PNAM has been focused on potential midface growth restrictions.^20–22^ Our findings are in line with those of Lee et al.,^20^ who suggested that PNAM does not alter growth. The absence of differences between the PNAM group and the NNAM group indicates that in the long-term growth is unlikely to be influenced by the technique used.

The results of this study should be viewed in the light of its limitations. Oral cleft treatment is a multistep procedure, and each step has its own effect on facial morphology. These effects cannot be separated from each other in a retrospective study.^21^ Furthermore, the outcome of oral cleft therapy depends upon the initial deformities.^23^ Although care was taken in this study to match the children in the NNAM and PNAM groups, this effect might make it difficult to determine which method is better. This is reflected by the high SD of the mean distance from the midline in the cleft groups, especially the PNAM group. PNAM has been shown to cause complications leading to poor parental compliance. Adverse effects such as irritation, rashes, and even inability of the parent to make the follow-up visits have all been listed as causes of low compliance.^24^ Although the PNAM group only included children who had completed the PNAM treatment before the surgery, it is impossible to predict parental compliance. This could explain a higher SD in the PNAM group when compared with the NNAM group. This study is also limited by the fact that photographs are a 2-dimensional representation of a 3-dimensional feature. Although techniques such as stereophotogrammetry have been proposed to overcome this limitation, many studies on facial symmetry in children with cleft lip and palate rely on photographic techniques.^12–14,16,17^

## CONCLUSIONS

PNAM does not seem to significantly impact long-term midface symmetry in children with CUCLP when compared with children treated without any form of presurgical infant orthopedics (PSIO).
